# Effects of an exercise intervention in primary care after robot-assisted radical cystectomy for urinary bladder cancer: a randomised controlled trial

**DOI:** 10.1186/s12885-024-12647-2

**Published:** 2024-07-24

**Authors:** Andrea Porserud, Patrik Karlsson, Markus Aly, Elisabeth Rydwik, Simon Torikka, Lars Henningsohn, Malin Nygren-Bonnier, Maria Hagströmer

**Affiliations:** 1 Division of Physiotherapy, Department of Neurobiology, Care Sciences and Society, Karolinska Institutet, Stockholm, Sweden; 2 Women’s Health and Allied Health Professionals Theme, Medical Unit Occupational Therapy & Physiotherapy, Karolinska University Hospital, Stockholm, Sweden; 3 Department of Molecular Medicine and Surgery, Karolinska Institutet, Stockholm, Sweden; 4 Patient Area Pelvic Cancer, Theme Cancer, Karolinska University Hospital, Stockholm, Sweden; 5 Division of Urology, Department of Clinical Science, Intervention and Technology, Karolinska Institutet, Stockholm, Sweden; 6 Academic Primary Health Care Centre, Region Stockholm, Stockholm, Sweden; 7 Department of Health Promoting Science, Sophiahemmet University, Stockholm, Sweden

**Keywords:** Cancer, Physical rehabilitation, Primary care, Quality of life, Surgery

## Abstract

**Introduction:**

After radical cystectomy physical activity is important to reduce risk of complications, but patients with urinary bladder cancer have difficulties in achieving general recommendations on physical activity and exercise. The aim of this randomised controlled trial was therefore to evaluate the effects of a physical exercise programme in primary care, following discharge from hospital after robot-assisted radical cystectomy for urinary bladder cancer.

**Materials and Methods:**

Patients with urinary bladder cancer scheduled for robot-assisted radical cystectomy at Karolinska University Hospital, Sweden between September 2019 and October 2022 were invited to join the study. At discharge, they were randomised to intervention or active control group. The intervention group was planned to start exercise with physiotherapist in primary care during the third week; the programme included aerobic and strengthening exercises, twice a week for 12 weeks, and daily walks. The control group received unsupervised home-based exercise with daily walks and a sit-to-stand exercise. Assessments were conducted before surgery, at discharge and after four months regarding the primary outcome physical function (Six-minute walk test), and secondary outcomes physical activity, pain, health-related quality of life, fatigue, and psychological wellbeing.

**Results:**

Ninety patients were included, mean (sd) age 71.5 (8.5) years. An intention-to-treat analysis showed no intervention effect on the primary outcome physical function, or on pain or psychological wellbeing, but effect on physical activity with a difference from discharge to four months with a median (IQR) of 4790 (3000) and 2670 (4340) daily steps in the intervention and control group, respectively (*p* = 0.046), and for fatigue, and health-related quality of life, in favour of the intervention group.

**Conclusion:**

Both the intervention and control groups improved physical function, but the patients who exercised in primary care experienced additional positive effects on physical activity, fatigue, and health-related quality of life. Hence, exercise in primary care after discharge from hospital could be a promising method after radical cystectomy for urinary bladder cancer.

**Trial registration:**

The study was registered in Clinical Trials with registration number NCT03998579, 20,190,607.

## Introduction

Physical activity is considered to decrease the risk of postoperative complications after surgery [1]. Radical cystectomy for urinary bladder cancer is high-risk surgery due to the considerable risk of postoperative complications. Readmission rates vary between 19 and 75% due to complications after robot-assisted radical cystectomy, for example infection, ileus, or thrombosis, which affect the patients’ recovery after surgery [2]. Patients who undergo radical cystectomy for urinary bladder cancer constitute a frail patient group with a mean age of 70 years, often suffering from comorbidity and malnutrition, and many of them are smokers [3]. It often takes up to six months for patients to recover and regain independence after a radical cystectomy [4]. For these patients, with a five-year overall survival rate of approximately 60% after the procedure, physical activity could decrease the risk of postoperative complications and improve physical function in the short term, and from a long-term perspective improve overall survival rates [1, 3, 5].

Regular physical activity has a positive impact on health, quality of life, and survival [6, 7]. For patients with cancer, there is strong evidence for the general benefits of physical activity, therefore physical activity is included in the concept of cancer rehabilitation. The recommendations are at least 150 min of moderate intensity physical activity per week and resistance exercise twice a week [8]. However, patients with urinary bladder cancer, like patients with other types of cancer, have displayed difficulties in achieving these levels of physical activity [9, 10]. After radical cystectomy, patients have expressed a need for more information about postoperative self-care, and specifically more information about physical activity [11]. However, information alone is potentially not enough for patients to reach the recommended levels of physical activity. Research on physical activity and exercise after radical cystectomy is limited; only one small study included in the systematic review evaluated an intervention early after discharge from hospital [12]. The aim of this study was therefore to evaluate the effects of a physical exercise programme in primary care, implemented early after discharge from hospital after robot-assisted radical cystectomy for urinary bladder cancer, regarding physical function, physical activity in daily life, pain, health-related quality of life, fatigue, and psychological wellbeing. Another aim was to evaluate whether patients had recovered to preoperative status four months after surgery.

## Materials and methods

### Study design

A single-blind, two-arm randomised controlled trial was conducted to evaluate the effects of a physical exercise programme. The intervention group exercised in the primary care centre and the control group (henceforth referred to as the active control group) was advised to perform a home-based exercise programme. The study was conducted in accordance with the Declaration of Helsinki, approved by the regional ethical review board in Stockholm (Dnr 2012/2214-31/4), registered in Clinical Trials (NCT03998579), and described in a study protocol [13]. The reporting is guided by the CONSORT extension for non-pharmacological treatments.

### Participants and settings

Patients with urinary bladder cancer were eligible if they were scheduled for a robot-assisted radical cystectomy at Karolinska University Hospital, Sweden between 1 September 2019 and 31 October 2022, and were consecutively invited to participate in the study. The patients underwent cystectomy with intracorporeal urinary diversion, either with an ileal conduit or an orthotopic bladder reconstruction [14]. The patients had to be able to speak and understand Swedish without an interpreter, be mobile with or without a walking aid, and live in Region Stockholm. Patients who were scheduled for palliative surgery or had a cognitive impairment defined in their medical records were not asked to participate in the study. Potential patients’ medical records were screened for eligibility by the researchers and eligible patients were given written information about the study by a registered nurse. After two or three days, the patients were contacted by a phone call from the researchers for further information. Informed consent was signed before the surgery. All patients received preoperative information on the importance of postoperative physical activity, and after surgery the Activity Board, which is a standardised tool involving individual goal-setting, was used on the ward to enhance mobilisation [15–17]. At discharge, patients were instructed to avoid lifting heavy objects and informed about the importance of physical activity at home.

For the exercise programme in primary care, 18 settings in Region Stockholm participated in the study. Hence, patients could choose to exercise in a primary care setting close to where they lived. A referral was sent from the physiotherapist at Karolinska University Hospital upon discharge from the hospital to the primary care setting the patient had chosen. Prior to the study’s start, the researchers educated the physiotherapists regarding the patient group, robot-assisted radical cystectomy, postoperative restrictions, the exercise programme, potential adverse events, and the study process.

### Intervention group

Patients who were randomised to an exercise programme with a physiotherapist in primary care were planned to start the exercise during the third week after discharge. The patients paid 200 SEK for their visits to primary care, in line with usual care. After the patients reached the high-cost threshold at 1400 SEK, the visits were free. The programme lasted for 12 weeks, with two exercise sessions per week, and was individually targeted but based on international recommendations for patients with cancer [8]. The programme consisted of aerobic exercise aiming for moderate intensity (30 min/session) and strengthening exercises for endurance training with 2 × 15 repetitions. The programme also included specific abdominal muscle training, including pelvic floor exercises, to minimise the risk of stoma hernia [18]. The exercise programme was approved by the surgeons who perform the cystectomies at Karolinska, and restrictions relating to abdominal muscles for six weeks due to surgical wounds were considered in the programme. Patients were also recommended to take daily walks. A recommended number of daily steps was set together with the physiotherapist once a week, based on the patient’s capacity the previous week. Individual goal setting, feedback, and self-monitoring of daily steps were used to support the patients’ daily walks.

### Control group

Patients who were randomised to the active control group, i.e., unsupervised home-based exercise, received written and oral instructions for a gradually increasing exercise programme that included daily walks and a sit-to-stand exercise. They also received information about supportive techniques to enhance their physical activity. Information about the exercise programme and supportive techniques was given on one occasion and the patients were not monitored regarding adherence or progression.

### Data collection and outcomes

Physiotherapists in primary care used exercise protocols to register the patients’ aerobic and strengthening exercises regarding intensity and weights. Adverse events were also registered in the protocols, with severe adverse events to be reported to the researchers. Demographic and clinical data was also collected from the patients’ medical records at Karolinska University Hospital.

The primary outcome was physical function, measured with the Six-minute walk test [19, 20]. Secondary outcomes were physical activity in daily life, i.e. daily steps, measured with the activity monitor activPAL3^®^ micro, which is a small device attached to the patient’s thigh with a bandage [21, 22], gait speed measured with maximum speed over six metres [23], as an indicator of leg strength the 30-second chair-stand test was used [24], grip strength measured with the Jamar dynamometer [25], pain measured with NRS [26], fatigue measured with the Piper Fatigue Scale [27], psychological wellbeing in terms of anxiety and depression measured with HADS [28], health-related quality of life measured with a cancer specific instrument EORTC QLQC30 [29], and EORTC BLM30 specific to muscle-invasive bladder cancer [30]. Details about how the measurements were conducted have previously been reported in the study protocol [13]. Patients performed physical tests and answered questionnaires on the day before surgery, at discharge from the hospital, and at four-month follow-up. Experienced physiotherapists conducted the tests on all occasions at Karolinska University Hospital. Physical activity was measured during the seven days after discharge and the seven days after the four-month follow-up.

### Sample size

Power was calculated on the primary outcome, physical function, measured with the Six-minute walk test. Based on a pilot study, we calculated an increased walking distance of 100 m in the intervention group and 70 m in the active control group, with a standard deviation of 30 m [31]. Thirty-two patients (16 in each group) would obtain a statistical power of 80% with type 1 error set at 0.05. Since the Six-minute walk test is highly correlated with age and sex, stratification by age and sex in the analysis was planned for, i.e., doubling the sample size. Due to this stratification and to guard against dropouts because of postoperative complications and readmission to hospital, it was planned to include 120 patients (60 in each group) in the study. Due to the COVID-19 pandemic and the length of data collection, a decision was taken to halt the inclusion at 100 patients.

### Allocation

Randomisation was conducted in blocks of 2–6 patients, stratified by sex and age (< 75, ≥ 75), through the ALEA computer system at the Centre for Clinical Cancer Studies at Karolinska University Hospital. A confirmation e-mail was sent to the researchers and an enrolment log was filed in the ALEA computer system. The patient received the next consecutive code number in the trial and treatment arm according to the randomisation scheme. After the tests at discharge from hospital, the physiotherapist received information from the researchers about which group the patient had been randomised to and informed the patient.

### Blinding

The ambition was that the physiotherapists conducting the measurements were to be blinded to the intervention. However, due to the pandemic and lack of resources in the clinics, not all measurements were blinded.

### Statistical analyses

Statistical analyses were conducted in SPSS version 28. Descriptive statistics were employed to ensure comparability between the groups prior to surgery and to describe the results of the outcomes. Due to non-normally distributed data and missing data, outcomes are described as median (IQR) and the non-parametric Paired Wilcoxon signed rank test was used to analyse differences between the measurements at discharge and four-month follow-up, per intervention group and control group, respectively. Mann-Whitney U-test was used to analyse effects between group differences regarding primary and secondary outcomes. The non-parametric Paired Wilcoxon signed rank test was also used to analyse differences between the measurements before surgery and at four-month follow-up, per intervention group and control group, respectively. Alpha level was set to 0.05 and data was analysed using both an intention-to-treat and per-protocol approach.

## Results

**Fig. 1:**
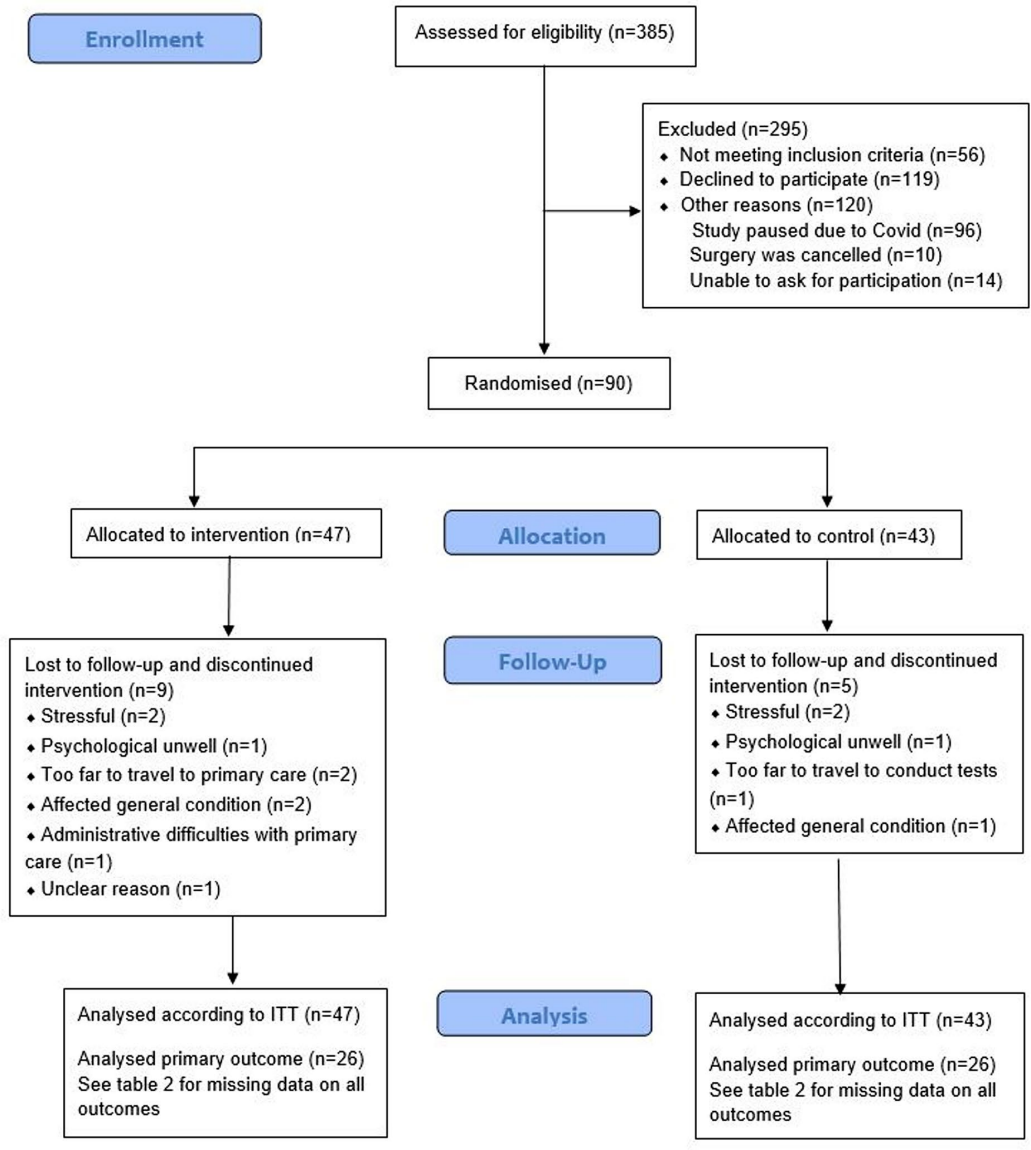
Flowchart of patients, analysed according to Intention to treat

Of 202 eligible and invited patients, 100 initially accepted but, due to dropouts during hospital stay, 90 (43%) patients were randomised at discharge. The mean (sd) age was 71.5 (8.5) years, 69% of the participating patients were men, and 83% had a robot-assisted radical cystectomy with an ileal conduit. In the intervention group, more patients had received treatment with Bacillus Calmette-Guérin (BCG) for urinary bladder cancer than in the control group. Apart from BCG treatment, there were no significant differences between the groups regarding preoperative demographic or clinical characteristics prior to the study start (see Table 1). Drop-out rates between tests at discharge and four-month follow-up were 19% in the intervention group and 12% in the control group (see Fig. 1). The rate of missing data for each measurement is presented in Table 2.

**Table 1:** Preoperative demographic and clinical characteristics of patients, presented as mean (sd)

	Intervention group *n* = 47		Control group *n* = 43	
Age, years	71.3	(9.1)	71.3	(7.8)
Women, n (%)	16	(34)	12	(28)
BMI, kg/m^2^	25.9	(4.6)	25.7	(4.5)
*Smoking status*,* n (%)*				
Smokers	2	(4)	4	(9)
Quit less than 6 months	3	(6)	6	(14)
Quit more than 6 months	29	(62)	18	(42)
Never smoked	13	(28)	15	(35)
*Family status*,* n (%)*				
Living alone	13	(28)	9	(21)
*Working status*,* n (%)*				
Working	10	(21)	10	(23)
Sick leave	3	(6)	1	(2)
Retired	34	(73)	32	(75)
*Preoperative clinical characteristics*				
Hypertension, n (%)	24	(51)	26	(60)
Diabetes, n (%)	7	(15)	9	(21)
*Tumour stage*,* n (%)*				
Ta	4	(9)	0	
T1	11	(23)	14	(33)
T2	31	(66)	27	(63)
T3	0		1	(2)
T4	1	(2)	0	
T not known	0		1	(2)
G1	1	(2)	0	
G2	1	(2)	4	(9)
G3	45	(96)	38	(89)
Gx	0		1	(2)
CIS	20	(43)	16	(37)
*Nephrostomy prior to surgery*,* n (%)*				
One	5	(11)	4	(9)
Two	0		1	(2)
*ASA class*,* n (%)*				
1	3	(6)	3	(7)
2	24	(51)	27	(63)
3	20	(43)	13	(30)
*Treatments before surgery*,* n (%)*				
Neoadjuvant chemotherapy	14	(30)	15	(35)
BCG	13	(28)*	4	(9)*
Previous abdominal surgery	12	(26)	15	(34)
*Surgery*				
Ileal conduit, n (%)	39	(83)	36	(84)
Orthotopic neobladder, n (%)	8	(17)	7	(16)
Operation time, minutes	275	(64)	301	(47)
*Postoperative clinical characteristics in hospital*				
Epidural, n (%)	1	(2)	1	(2)
Time to first stool, days	3.9	(1.5)	3.9	(1.4)
Temperature over 38.5, n (%)	4	(9)	4	(9)
Ventricle drain, n (%)	14	(30)	9	(21)
Reoperation, n (%)	3	(6)	1	(2)
Length of stay at hospital, days	8.5	(3.7)	8.9	(6.7)
Discharged to rehabilitation ward, n (%)	32	(68)	23	(53)

*Significant difference between the groups, Gx = Squamous epithelial, CIS = Cancer in situ, ASA = American Society of Anesthesiologists, BCG = Bacillus Calmette-Guérin

**Table 2:** Missing data, presented as numbers and proportion, n (%) of each measurement

		Intervention group *n* = 47			Control group *n* = 43		
		Day before surgery	Discharge (Baseline)	4-month follow-up	Day before surgery	Discharge (Baseline)	4-month follow-up
**Variable**	***Instrument***						
Physical function	*6 MWT*	3 (6)	12 (25)	21 (45)	6 (14)	9 (21)	17 (39)
Physical activity, daily steps	*activPAL*		9 (19)	18 (38)		6 (14)	13 (30)
Gait speed	*Walk test 6 m max*	3 (6)	11 (23)	21 (45)	5 (12)	7 (16)	17 (39)
Leg strength	*30 s chair stand test*	3 (6)	8 (17)	21 (45)	3 (7)	6 (14)	17 (39)
Grip strength	*Jamar dynamometer*	3 (6)	7 (15)	21 (45)	5 (12)	5 (12)	17 (39)
Pain	*NRS*	3 (6)	7 (15)	21 (45)	3 (7)	6 (14)	17 (39)
Fatigue	*PFS*	3 (6)	2 (4)	15 (32)	4 (9)	1 (2)	11 (26)
Depression	*HADS*	4 (9)	2 (4)	15 (32)	4 (9)	1 (2)	11 (26)
Anxiety	*HADS*	4 (9)	2 (4)	15 (32)	4 (9)	1 (2)	11 (26)
HRQoL global health	*EORTC QLQC30*	3 (6)	2 (4)	15 (32)	5 (12)	2 (5)	11 (26)
HRQoL total function	*EORTC QLQC30*	3 (6)	2 (4)	15 (32)	5 (12)	2 (5)	11 (26)
HRQoL total symptoms	*EORTC QLQC30*	3 (6)	2 (4)	15 (32)	5 (12)	2 (5)	11 (26)
HRQoL bladder cancer specific symptoms	*EORTC BLM30*	3 (6)	2 (4)	15 (32)	4 (9)	1 (2)	11 (26)

HRQoL = Health-related quality of life

**Fig. 2:**
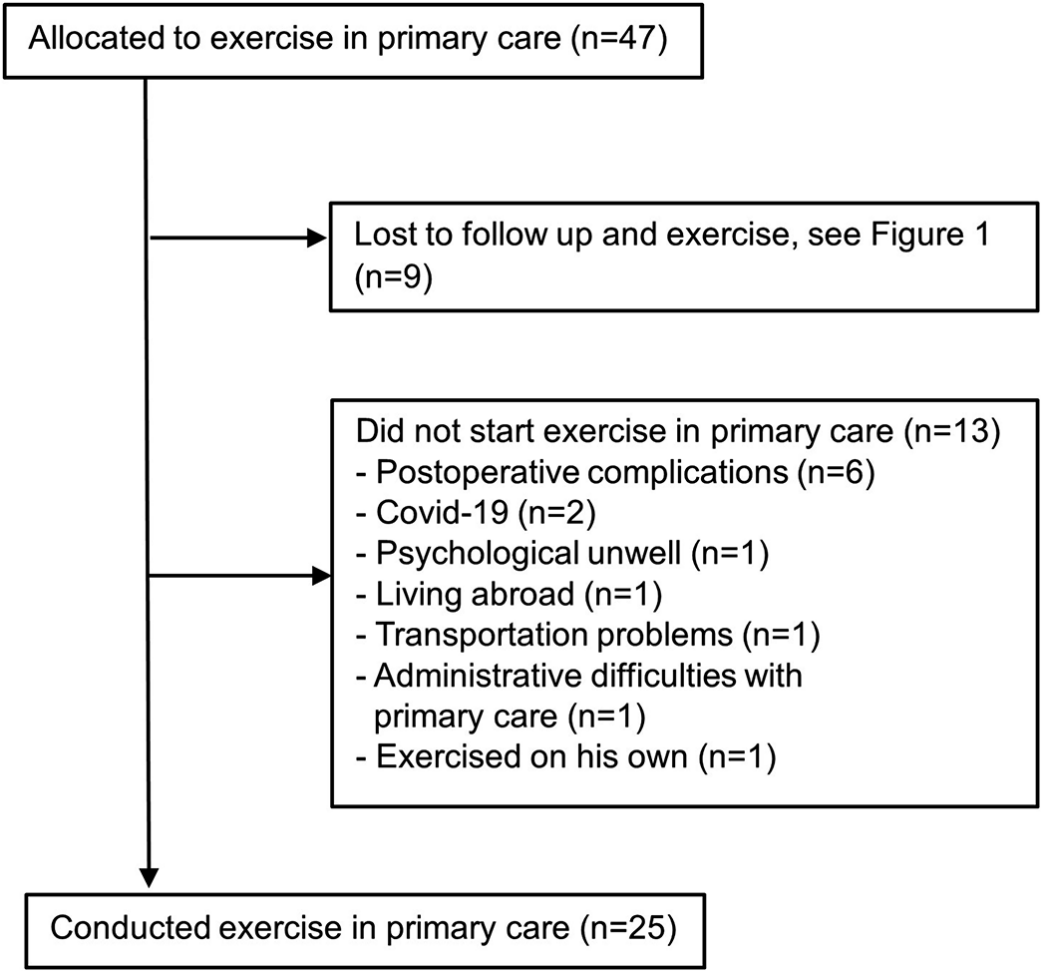
Flowchart of patients allocated to exercise in primary care

Physiotherapists in the primary care centres registered the exercise sessions conducted for the intervention group in an exercise protocol. In total, 25 of the 47 patients included in the intervention group started the exercise in primary care (see Fig. 2). The patients had their first exercise session a mean (sd) of 39 (21) days after discharge from hospital, with 25% within three weeks of discharge. Patients participated in a mean (sd) of 17 (6) exercise sessions. No adverse events during the exercise in primary care were registered in the study protocols or reported to the researchers.

Table 3 describes the outcomes and within group differences at discharge (baseline) and four-month follow-up as well as the effect of between group differences for primary and secondary outcomes. The results showed no intervention effect on the primary outcome of physical function, but a positive intervention effect (*p* < 0.05) on the secondary outcomes of daily steps, fatigue, and health-related quality of life (global health, total symptoms, and bladder-cancer-specific symptoms). A per-protocol analysis was also conducted and included patients who had attended at least one third (eight exercise sessions) of the programme. The per-protocol analysis revealed the same result as the intention-to-treat analysis.

**Table 3:** Description of outcomes and differences between outcomes at discharge and 4-month follow up

	Intervention					Control					Intervention effect				
Outcome variable	Discharge (Baseline)		4-month follow-up		*P*- value*	Discharge (Baseline)		4-month follow-up		*P*-value*	Intervention (difference)		Control (difference)		*P*-value**
Physical function, m	376	(120)	490	(129)	**< 0.001**	370	(97)	515	(132)	**< 0.001**	131	(152)	157	(104)	0.762
Physical activity, daily steps, n	2810	(2390)	7400	(4650)	**< 0.001**	2640	(2980)	7060	(3830)	**< 0.001**	4790	(3000)	2670	(4340)	**0.046**
Gait speed, m/s	1.06	(0.40)	1.60	(0.54)	**0.001**	1.12	(0.54)	1.58	(0.44)	**< 0.001**	0.59	(0.48)	0.48	(0.51)	0.324
Leg strength, n	8	(4.5)	13	(7)	**< 0.001**	9	(5)	12	(6.5)	**< 0.001**	6	(5)	4	(5)	0.671
Grip strength^a^, kg	31.6	(14.6)	34.4	(12.2)	0.108	30.6	(13.7)	37.1	(12.1)	0.117	1.6	(2.9)	1.5	(3.5)	0.781
Pain (0–10)^b^	1	(4)	0	(0)	**0.013**	1	(3)	0	(1)	0.357	-2	(2.8)	-1	(3)	0.205
Fatigue (0–10)^b^	6	(3)	1	(4)	**< 0.001**	6	(3)	4	(5.3)	**< 0.001**	-4	(3)	-3	(3)	**0.008**
Depression^b^ (0–21)	5	(7)	1.5	(4)	**< 0.001**	6	(4)	4	(4.3)	0.058	-3	(4.5)	-1	(5.3)	0.084
Anxiety^b^ (0–21)	6	(6)	2.5	(5)	**< 0.001**	7	(5)	4	(6)	0.123	-2	(5)	-1.5	(3.3)	0.065
HRQoL global health (0-100)	42	(33)	83	(33)	**< 0.001**	33	(33)	67	(33)	**< 0.001**	42	(29)	33	(45.5)	**0.024**
HRQoL total function (0-100)	47	(28)	87	(20.5)	**< 0.001**	51	(22)	78	(25.3)	**< 0.001**	36	(24)	26	(29.5)	0.050
HRQoL total symptoms (0-100)^b^	54	(28)	13	(13.5)	**< 0.001**	51	(28)	18	(13.8)	**< 0.001**	-43	(18.5)	-33	(28.5)	**0.025**
HRQoL specific symptoms^c^ (0-100)^b^	33	(22)	18	(24.5)	**< 0.001**	36	(20.5)	26	(21.8)	**0.003**	-22	(18.5)	-13.5	(26.5)	**0.042**

Presented as median (IQR) and effect of within group and between group differences

*Paired Wilcoxon signed rank test, **Mann-Whitney U-test, ^a^Mean value of right and left hand together, ^b^ Higher score means more symptoms, ^c^ HRQoL bladder cancer specific symptoms, HRQoL = Health related quality of life

Table 4 presents the within group patients’ recovery at four-month follow-up. Both groups had regained their preoperative values, with no significant differences between measurements before surgery and at four-month follow-up for most of the measurements. However, four months after surgery, only the intervention group had improved the scores of depression, anxiety, and health-related quality of life (global health, total function, and bladder-cancer-specific symptoms) compared to preoperative values.

**Table 4:** Descriptives of the outcomes and effect of within group differences between tests

Outcomes	Intervention group *n* = 47			Control group *n* = 43		
	Day before surgery	4-month follow-up	P-value^*^	Day before surgery	4-month follow-up	P-value^*^
Physical function, m	491 (173)	490 (129)	0.092	492 (194)	515 (132)	0.939
Gait speed, 6 m max, m/s	1.51 (0.405)	1.60 (0.538)	0.368	1.71 (0.520)	1.58 (0.440)	0.626
Leg strength, n	12 (6.0)	13 (6.75)	0.541	12.5 (5)	12 (6.5)	0.760
Grip strength, kg	36.4 (13.0)	34.4 (12.2)	0.145	33.0 (14.3)	37.1 (12.1)	0.140
Pain (0–10)^a^	0 (0.25)	0 (0)	0.573	0 (0)	0 (1)	0.434
Fatigue (0–10)^a^	0.5 (5)	1 (4)	0.507	4 (5)	4 (5.25)	0.795
Depression (0–21)^a^	3 (3.5)	1.5 (4)	**0.037**	4 (4)	4 (4.25)	0.875
Anxiety (0–21)^a^	6 (5)	2.5 (5)	**< 0.001**	6 (4)	4 (6)	0.888
HRQoL global health (0-100)	67 (33)	83 (33)	**0.002**	67 (31)	67 (33)	0.328
HRQoL total function (0-100)	80 (23.3)	87 (20.5)	**0.002**	82 (14)	78 (25.3)	0.715
HRQoL total symptom (0-100)^a^	15 (15)	13 (13.5)	0.268	15 (20.3)	18 (13.8)	0.522
HRQoL (bladder cancer specific) (0-100)^a^	29 (20.5)	18 (24.5)	**0.011**	29 (14.5)	26 (21.8)	0.254

Presented as median (IQR),^*^Paired Wilcoxon signed rank test, ^a^ Higher score means more symptoms, HRQoL = Health-related quality of life

## Discussion

This single-blind, RCT involving individuals who have undergone robot-assisted radical cystectomy for urinary bladder cancer investigated the effects of an exercise programme in primary care in terms of physical function, physical activity in daily life, and psychological wellbeing. We used an active control group that was recommended to exercise at home, and the intention of blinding the assessors. We did not find a significant group difference for the primary outcome, the Six-minute walk test. However, a significant group difference was found for daily steps, fatigue, and health-related quality of life for global health, total symptoms, and bladder-cancer-specific symptoms, in favour of the intervention group.

Studies that evaluate physical exercise early after abdominal cancer surgery in general, and radical cystectomy specifically are scarce. There is however strong evidence in systematic reviews that supervised exercise programmes have positive effects on physical function, fatigue, health-related quality of life, depression, and anxiety during or after other cancer treatments [8, 32, 33]. A review of aerobic exercise after abdominal cancer surgery concluded that exercise is also valuable for these patients for improving physiological and psychological parameters but requested larger studies in the future [34]. This present study is one of the first to show that a supervised exercise programme has positive effects on physical activity in daily life, fatigue, and health-related quality of life soon after robot-assisted radical cystectomy.

The primary outcome, physical function, did not show any between group differences, perhaps because daily walking was the main exercise component for the active control group. However, a systematic review evaluating efficacy on prehabilitation and/or rehabilitation after radical cystectomy showed improvements for the intervention groups in physical function, in two studies measured with the Six-minute walk test [12]. The patients who were allocated to control groups in the systematic review all received standard treatment, i.e. no sorts of exercise program, which instead was the case for the active control group in this study, resulting in no between group differences on physical function. The intervention group, on the other hand, did increase the number of daily steps more than the control group after the intervention period. Potentially, the whole-body exercise programme may therefore result in more general daily physical activity, instead of just taking walks. Although the patients who exercised in primary care increased their number of steps more than the control group, both groups were taking more than 7000 steps per day, as a median, at the four-month follow-up. One important aspect is that both the intervention and control group in this study were given postoperative exercise programmes to follow, which seem to have positive effects.

For the patients randomised to exercise in primary care, most domains of cancer-specific health-related quality of life had improved more after 12 weeks of exercise than among the control group. However, the previous suggestion of differences of ≥ 10 points as clinically relevant for all scales in the questionnaire EORTC QLQC30 has recently been evaluated and is now recommended to be used with caution since it could differ between scales in the questionnaire and between different cancer diagnoses [35]. Two reviews have previously summarised prehabilitation and rehabilitation effects on health-related quality of life after radical cystectomy [12, 36]. Only two studies that evaluated physical rehabilitation were included in the reviews, evaluating postoperative rehabilitation at the hospital ward, and exercise after discharge but conducted at the hospital, [31, 37]. Both studies showed effect on a few of the domains in health-related quality of life, but as the reviews conclude the evidence has been scarce. Potentially in this study, the assistance of a physiotherapist could strengthen patients’ self-efficacy regarding exercise and physical activity, and result in better health-related quality of life. According to qualitative studies, patients who undergo radical cystectomy experience information on exercise and physical activity as too limited and vague at discharge from hospital [11]. Not having information also led to fear and thus avoidance of physical activity and exercise, and potentially also a negatively affected health-related quality of life [38, 39]. Patients complained of a lack of information about what type of rehabilitation could enhance their recovery [11].

All the patients included in this study received information on rehabilitation, in either primary care or unsupervised home-based exercise. Both groups had recovered to preoperative values at four-month follow-up. This result is in line with a recent study in which patients who underwent radical cystectomy had recovered three to six months after surgery, with respect to activities in daily life, functional capacity measured with timed up and go, and hand grip strength [4]. However, the present study also showed that the patients who had exercised in primary care together with a physiotherapist felt less depressed, less anxious, and rated their health-related quality of life as better four months after surgery than it had been before surgery. This within group analysis adds information to the between group analysis since depression and anxiety are important aspects addressed by cancer rehabilitation.

### Limitations

Due to the COVID-19 pandemic, the study was paused on several occasions because the patients who were randomised to intervention could not exercise in primary care. Also, the patients could not visit the hospital for assessments. Thereby, it was not possible to conduct any physical tests during these periods; instead, patients filled in questionnaires at home and sent them by mail which resulted in missing data, mainly at the four-month follow-up. The intention that the physiotherapists who conducted the tests should be blinded was not completely fulfilled as some patients were eager to talk about the exercise. In addition, since the physiotherapists sometimes had limited resources, the researchers conducted some tests. The pauses caused a prolonged inclusion period that was ended at 100 patients instead of 120, one year after the original time plan had expired. Postoperative complications or psychological impairment related to surgery and postoperative recovery were the most common reasons to not begin exercise in primary care or to drop-out from the study. Although sample size was calculated as a total of 32 patients for the intervention to have effect on the primary outcome of physical function, measured with the Six-minute walk test, no intervention effect was seen. One reason could be that daily walks were the main exercise component for the active control group.

## Conclusion

Both the intervention and control groups improved physical function, but the patients who exercised in primary care experienced additional positive effects on physical activity, fatigue, and health-related quality of life. Hence, exercise in primary care including aerobic and strengthening exercises at a moderate intensity after discharge from hospital could be a promising method after radical cystectomy for urinary bladder cancer.

## Data Availability

The datasets generated during and/or analysed during the current study are not publicly available due to Swedish and EU personal data legislation but are available from the corresponding author on reasonable request. Any sharing of data will be regulated via a data transfer and user agreement with the recipient.

## Abbreviations

ASA: American Society of Anesthesiologists
BCG: Bacillus Calmette-Guérin
BMI: Body Mass Index
CIS: Cancer in situ
HADS: Hospital Anxiety Depression Scale
HRQoL: Health-Related Quality of Life
m/s: Meters Per Second
NRS: Numeric Rating Score
RCT: Randomised Controlled Trial
sec: Seconds
SEK: Swedish Crowns
